# Percutaneous achillotomy in the treatment of congenital clubfoot: should it be performed in the operating theater or the polyclinic?

**DOI:** 10.1186/s13018-018-0851-9

**Published:** 2018-06-19

**Authors:** Ümit Tuhanioğlu, Hasan U. Oğur, Fırat Seyfettinoğlu, Hakan Çiçek, Volkan T. Tekbaş, Ahmet Kapukaya

**Affiliations:** Department of Orthopaedics and Traumatology, University of Health Science Adana City Hospital, Adana, Turkey

**Keywords:** Ponseti, Achillotomy, Clubfoot

## Abstract

**Background:**

The aim of this study was to compare the efficacy, advantages, and complications of percutaneous achillotomy in the treatment of clubfoot with the Ponseti method when performed to two different groups under general anesthesia or polyclinic conditions with local anesthesia.

**Methods:**

A retrospective evaluation was made of 96 patients treated for clubfoot in our clinic between January 2013 and June 2016. Fifty-seven patients were separated into two groups according to whether the achillotomy was performed in polyclinic conditions with local anesthesia or under general anesthesia following serial plaster casting with the Ponseti method.

**Results:**

The characteristics of age distribution, mean week of tenotomy, side, and sex were similar in both groups. No statistically significant difference was determined between the two groups in respect to complication and recurrence. The durations of hospitalization-observation, separation from the mother, and fasting were found to be statistically significantly shorter in local anesthesia group.

**Conclusion:**

Although the performance of percutaneous achillotomy with local or general anesthesia has different advantages, it can be considered that especially in centers with high patient circulation, achillotomy with local anesthesia can be more preferable to general anesthesia because it is practical and quick, does not require a long period of fasting or hospitalization, and has a similar complication rate to general anesthesia procedures.

## Background

Congenital clubfoot deformity is a congenital deformity that can be successfully treated with current conservative methods, and when not treated, can cause severe pain and functional impairments [1]. It is seen at the rate of 5 in 10,000 births [2]. The Ponseti method is a widely accepted and used method for the treatment of congenital clubfoot. As positive results have been reported with this method in extensive series and long-term follow-up studies, it has been accepted by many authors as the gold standard in congenital clubfoot treatment [3–8]. The Ponseti method is a comprehensive treatment method in which cavus, adduction, and heel varus deformities are corrected with a series of plaster casts, and in most cases, there is a need for intervention for the equinus with a percutaneous achillotomy. Finally, to protect the correction, there is need for long-term use of a brace [8]. Although the Ponseti method seems to be simple in application, there are sensitive points such as manipulating the plaster cast, percutaneous achillotomy, and the use of a brace. Incorrect application can result in relapse and complex clubfoot deformities. Many studies have reported complications and recurrence associated with problems in treatment adherence and incomplete or erroneous application of the principles [9–12].

Percutaneous Achilles tenotomy (PAT) is an important component of clubfoot treatment with the Ponseti method [13]. Just as there are authors who have reported the application of PAT in the polyclinic with local anesthesia [14], there are also studies that have stated that the procedure is safer and more comfortable when performed in the operating room [15].

The aim of this study was to compare the efficacy, advantages, and complications of percutaneous achillotomy in the treatment of clubfoot with the Ponseti method when performed with two different groups under general anesthesia or in the polyclinic with local anesthesia.

## Methods

A retrospective evaluation was conducted of 96 patients treated for clubfoot in our clinic between January 2013 and June 2016. A total of 83 achillotomies (86%) were performed to these patients. A total of 26 patients were excluded from the study due to previous treatment, neuromuscular etiology, or withdrawal from follow-up. The remaining 57 patients were separated into two groups according to whether the achillotomy was performed in the polyclinic with local anesthesia (group 1, 32 patients, 47 feet) or under general anesthesia (group 2, 25 patients, 38 feet) following serial plaster casting with the Ponseti method. The patients in both groups were evaluated after the achillotomy after obtaining 70° abduction with repeated correction casting applied to the clubfoot. Patients with < 15° dorsiflexion in the ankle determined at the clinical examination had percutaneous achilloplasty planned.

**Fig. 1:**
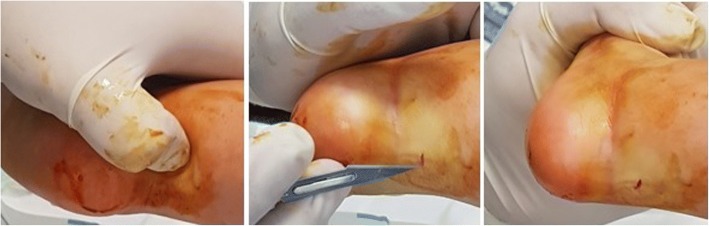
Palpation of Achilles tendon with nail tip, percutaneous Achilles tenotomy, and increase in dorsiflexion with a click sensation

In the procedures performed with local anesthesia, the infants were fasted for 1–2 h before the procedure. As the procedure started, bottle-feeding was started by an assistant. The purpose of bottle-feeding the infant during the procedure was to distract the infant’s attention so they would feel less pain and cry less. Then, local anesthesia was applied as subcutaneous lidocaine hydrochloride that did not exceed a total dose of 1.5 mg/kg. The percutaneous achillotomy was performed from 1 cm proximal to the Achilles tendon attachment site and from the medial border of the Achilles tendon. A sudden increase in dorsiflexion with a clicking indicated that the achillotomy was complete (Fig. 1). A long-leg plaster cast was applied for 3 weeks with the foot at 70° abduction and 15° dorsiflexion. Immediately after the procedure, 10 mg/kg acetaminophen was administered rectally, and the mother breastfed the infant. After 1 h of follow-up, the patient was discharged home with any necessary recommendations.

The achillotomy was performed to the patients in group 2 with the same technique but under general anesthesia. Before general anesthesia, infants are fasted for 4–6 h depending on age, and feeding was permitted 2 h postoperatively. Postoperative analgesia was provided in the same way as for group 1. Patients were hospitalized for one night and discharged the following day.

At the final follow-up examination, the patients were compared on anamnesis, observational and physical examination findings, detailed information from the family, the number of plaster casts, recurrence, complications, duration of hospitalization for observation, duration of separation of the infant from the mother, and duration of fasting.

The ethical research committee approved the study protocol (ANEAH/13.9.2017-99). Written consent was obtained from the patient’s next-of-kin.

## Results

A total of 57 patients (85 feet) were included in the two groups in this study. Group 1 comprised 22 males and 10 females with a mean age at the start of treatment of 12.3 weeks, and group 2 consisted of 16 males and 9 females with a mean age of 11.5 weeks. In group 1, 47 feet of 32 patients underwent percutaneous achillotomy in the polyclinic with local anesthesia, and in group 2, 38 feet of 25 patients received percutaneous achillotomy under general anesthesia.

The age distribution, mean weeks of tenotomy, side, and sex were similar in both groups (Table 1).

**Table 1:** Age, gender, side, and tenotomy week of the groups

		*n*	Mean	Median	Minimum	Maximum	SD
Age (weeks)	LA group	32	12.3	3.0	1	36	22.8
	GA group	25	11.5	4.0	1	44	24.6
Tenotomy (week)	LA group	47	5.9	6.0	4	7	0.9
	GA group	38	5.8	6.0	5	8	0.6
		Group					
		L A		G A			
		*n*	%	*n*		%	
Side	Left	7	14.9	6		15.8	
	Right	8	17.0	6		15.8	
	Bilateral	16	68.1	13		68.4	
	Total	47	100.0	38		100.0	
Gender	Male	22	68.7	16		64	
	Female	10	31.3	9		36	
	Total	32	100.0	25		100.0	

*GA* general anesthesia, *LA* local anesthesia, *SD* standard deviation

The complication of bleeding was seen in 2 feet (4.2%) in the local anesthesia group and in 1 foot (2.6%) in the general anesthesia group, which was not significantly different (*p* = 0.398). Recurrence was seen in five patients (10.6%) in the local anesthesia group and in three patients (8.1%) in the general anesthesia group at the end of a mean follow-up of 28.3 months. The reasons for recurrence were determined to be incomplete achillotomy in one patient and non-adherence with orthosis in four patients in the local anesthesia group. In group 2, the reason in all three patients was non-adherence with orthosis. No cases of incomplete achillotomy were found in group 2. No significant difference was found between the two groups with respect to recurrence (*p* = 0.985) (Table 2). No infection, impairment in circulation, or anesthesia-related complications were seen in any patients in either group.

**Table 2:**
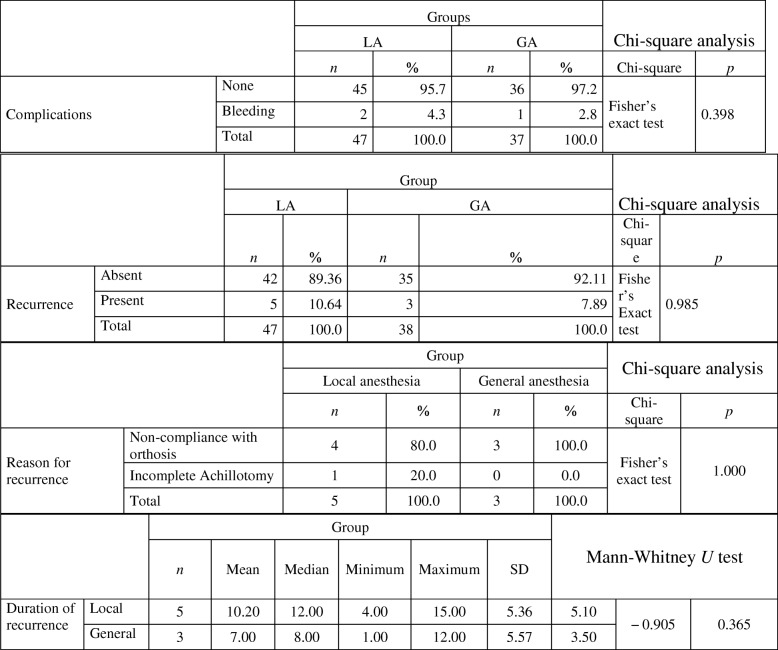
Complications and recurrence (reason, distributions, duration)

The duration of hospitalization for observation, separation from the mother, and fasting were found to be significantly shorter in group 1 (*p* < 0.05) (Table 3).

**Table 3:** Hospitalization-observation, separation from mother, fasting periods, operative and casting time in the groups

		Duration of hospitalization and observation (h)						Mann-Whitney *U* test		
		*n*	Mean	Median	Minimum	Maximum	SD			
Groups	Local anesthesia	32	1.41	1.00	.75	11.00	1.77	16.50	− 6.44	0.0001
	General anesthesia	25	26.13	24.00	19.00	60.00	9.17	44.50		
		Duration of separation of the infant from the mother (min)						Mann-Whitney *U* test		
		*n*	Mean	Median	Minimum	Maximum	SD			
Groups	Local anesthesia	32	12.3	12	6	20	3.4	16.00	− 6.3	0.0001
	General anesthesia	25	53.5	55	40	70	9.1	43.50		
		Fasting period before procedure (h)						Mann-Whitney *U* test		
		*n*	Mean	Median	Minimum	Maximum	SD			
Groups	Local anesthesia	32	1.34	1	1	2	0.48	16.5	6.70	0.0001
	General anesthesia	25	4.88	4	4	6	1.01	45		
		Operative time and casting time						Mann-Whitney *U* test		
		*n*	Mean	Median	Minimum	Maximum	SD			
Groups	Local anesthesia (casting time)	32	15	13	8	25	3.8	24.00	− 5.2	0.06
	General anesthesia (operative time)	25	26	27	18	32	4.1	38.50		

## Discussion

Pes equinovarus is a complex, three-dimensional foot deformity, characterized by cavus and aductus of the forefoot and varus and equine deformity of the hindfoot [4]. In comparison with conservative and surgical methods used in the past, the Ponseti method has achieved a high success rate and is a relatively non-invasive treatment. It has come into orthopedic practice as a widely accepted treatment for clubfoot.

The Ponseti method aims to correct the deformity with weekly manipulations, serial plaster casting, and remodeling of the joint, and after the serial plaster casting, a percutaneous achillotomy is performed with a minimally invasive technique, and finally, a brace is used. The achillotomy is required in approximately 80–90% of infants with clubfoot [16, 17]. Achillotomy in patients with clubfoot is generally made percutaneously because it results in minimal fibrosis and scar formation. Some surgeons prefer to perform achillotomies under general anesthesia with a mini-incision to visualize the Achilles tendon, with the aim of protecting vessels and nerves from injury that can occur during percutaneous achillotomy and to avoid incomplete achillotomy [18]. Nevertheless, the procedure is generally performed as a percutaneous achillotomy under general or local anesthesia [19–21]. The procedure performed under sedation and general anesthesia has been reported to have the advantages of reducing complications, providing a complete achillotomy, being a more controlled procedure, and providing analgesia [19, 20, 22]. In these studies, it was reported that the general anesthesia was safer for the intervention and better at preventing major complications from surgery. Parada et al. performed 89 percutaneous achillotomies under general anesthesia and reported no complications [15]. In contrast, Ponseti himself performed percutaneous achillotomy in an environment outside of the operating theater [22]. Lebel et al. applied percutaneous achillotomy with local anesthesia as an office and described it as safe and effective [14]. Some studies have reported severe vascular bleeding, pseudoaneurysm, and combined vascular and nerve injuries following percutaneous achillotomy performed under general anesthesia [7, 23, 24]. The bleeding occurring after the percutaneous achillotomy in these studies was reported to have been stopped with compression. In the current study, bleeding occurred in 3 feet (4.2%) in the local anesthesia group and in 1 foot (2.6%) in the general anesthesia group. By applying compression, the bleeding in the patients in both groups was brought under control, and then the skin was sutured. In the subsequent period, no complications developed related to circulatory problems, such as infection or hematoma. With the percutaneous achillotomy technique, when the Achilles tendon and surrounding vessels and nerve structures cannot be directly visualized, it is vulnerable to these types of complications. In the current study, no differences were found between the groups with respect to complication rates. These types of complications are related to the technique, and the evaluation between the two groups was made independent of the location of the procedure or the type of anesthesia used. Furthermore, as compression was applied for bleeding in both groups, the results suggest that there was no superiority for general anesthesia over local anesthesia.

If the infant feels pain during local anesthesia, it can be an upsetting and worrying state for both the doctor and the family. However, when the correct dose is administered in the correct location, pain is usually limited to the prick of the fine anesthesia needle. On the other hand, in procedures such as taking blood and opening a vascular route in the preoperative preparation for general anesthesia, the infant will feel pain, and most of the time, it is a fact that they will be exposed to repeated interventions.

In the current study, the duration of fasting for the infant was statistically significantly longer in the general anesthesia group. It was observed that 6–8 h fasting for the infant was difficult and distressing for the family and the infant, and when a retrospective examination was made, it was noticeable that all the families clearly remembered the time that the infant remained hungry. In this respect, local anesthesia was tolerated better than general anesthesia by the infant and the family.

When the duration of hospitalization and observation were compared, the duration of hospitalization and observation in group 2 was seen to be statistically significantly longer. Especially in centers such as ours where there are large numbers of patients, and the number of beds and operating room conditions cannot respond sufficiently to the volume, achillotomy with local anesthesia is preferred because it will contribute to reducing the intensity in the wards and the operating theaters.

Studies have reported dose-related anaphylaxis and arrest following local anesthesia. However, there are no reports in the literature of systemic complications after achillotomy performed with local anesthesia. In the current series, no anaphylaxis and cardiac or neurological complications related to local anesthesia were encountered. However, to be able to intervene in these types of complications, care should be taken as to the location of the local anesthesia supplies for emergency interventions and from which the emergency pediatric and anesthesia units can easily and rapidly reach. In the current series, the room where the percutaneous achillotomy was performed under local anesthesia was equipped for emergency intervention before each case and was on the same floor as the Emergency Pediatric and Anesthesia Units. In addition, great care was taken not to exceed the safe dose for local anesthesia (lidocaine < 1.5 mg/kg). Despite all the precautions and appropriate equipment, the families were informed before the procedure of complications that could develop, and an informed consent form was signed.

The duration of separation of the infant from the mother was found to be statistically significantly longer in the general anesthesia group. As the duration of separation increases, there is extra worry and stress for both the mother and infant, which has a negative psychological effect on both individuals. As a general observation, although it was predicted that the stress and worry level of the families of the patients in group 2 would be at a subjectively higher level, it was not possible to obtain objective data retrospectively. It is thought that in similar prospective studies, the measurement of worry and anxiety with objective tests before the procedure could be significant.

Cost is another important parameter in selecting procedures that provide similar results. However, a comparison of costs could not be made in this study because the costs of procedures in our center are made independently of the type of anesthesia. However, in different centers, there could be different costs for achillotomies performed under local or general anesthesia, and in this context, cost comparisons should be made in future studies.

Incomplete achillotomy is a potential complication in patients with achillotomy performed under local anesthesia. Authors who prefer general anesthesia have reported that they feel safer and more in control of the procedure. It has been emphasized that as a result of the procedure being performed with general anesthesia in operating room conditions, incomplete achillotomy can be avoided, and there is less formation of rigid equinus and rocker bottom deformities [18–20]. In studies where the procedure was performed with local anesthesia, low rates of incomplete achillotomy and associated recurrence and deformity have been reported [23]. In the current study, although incomplete achillotomy and associated persistent hindfoot equinus and recurrence were seen in one patient in group 1, no incomplete achillotomy was found in group 2. As the sample size was small, no statistical evaluation could be made in respect to incomplete Achillotomy and recurrence of the deformity. A limitation was that the negative outcomes of incomplete Achillotomy and recurrence as a result of the procedure applied with local anesthetic could not be sufficiently evaluated because of the short follow-up period. Therefore, in a longer follow-up period when a difference emerges between the two groups in respect to incomplete Achillotomy and recurrence rates, a stronger recommendation could be made on the subject of Achillotomy under polyclinic conditions.

## Conclusion

Although percutaneous achillotomy with local or general anesthesia has different advantages, it can be considered that, especially in centers with high patient circulation, achillotomy with local anesthesia can be more preferable to general anesthesia because it is practical and quick, does not require a long period of fasting or hospitalization, and has a similar complication rate to general anesthesia procedures. Nevertheless, it is of critical importance that the area of application is sterile, safe, and comfortable and that the family is informed in detail and provides informed consent.

## Abbreviation

PAT: Percutaneous Achilles tenotomy
